# The N-terminal domains of FLASH and Lsm11 form a 2:1 heterotrimer for histone pre-mRNA 3’-end processing

**DOI:** 10.1371/journal.pone.0186034

**Published:** 2017-10-11

**Authors:** Wei Shen Aik, Min-Han Lin, Dazhi Tan, Ashutosh Tripathy, William F. Marzluff, Zbigniew Dominski, Chi-Yuan Chou, Liang Tong

**Affiliations:** 1 Department of Biological Sciences, Columbia University, New York, New York, United States of America; 2 Department of Life Sciences and Institute of Genome Sciences, National Yang-Ming University, Taipei, Taiwan; 3 Department of Biochemistry and Biophysics, University of North Carolina at Chapel Hill, Chapel Hill, North Carolina, United States of America; Korea University, REPUBLIC OF KOREA

## Abstract

Unlike canonical pre-mRNAs, animal replication-dependent histone pre-mRNAs lack introns and are processed at the 3’-end by a mechanism distinct from cleavage and polyadenylation. They have a 3’ stem loop and histone downstream element (HDE) that are recognized by stem-loop binding protein (SLBP) and U7 snRNP, respectively. The N-terminal domain (NTD) of Lsm11, a component of U7 snRNP, interacts with FLASH NTD and these two proteins recruit the histone cleavage complex containing the CPSF-73 endonuclease for the cleavage reaction. Here, we determined crystal structures of FLASH NTD and found that it forms a coiled-coil dimer. Using solution light scattering, we characterized the stoichiometry of the FLASH NTD-Lsm11 NTD complex and found that it is a 2:1 heterotrimer, which is supported by observations from analytical ultracentrifugation and crosslinking.

## Introduction

In eukaryotic cells, histones play important roles in genomic DNA packaging as well as epigenetic regulation of gene expression. The levels of histone mRNAs are carefully controlled throughout the cell cycle and they dramatically increase during S phase to meet the growing demand for packaging the newly replicated DNA from the replicating genome [1]. In metazoans, histone proteins for packaging of newly synthesized DNA are encoded by the replication-dependent histone genes. They are distinct from the replication-independent histone genes which are expressed constitutively [2]. Unlike canonical pre-mRNAs, replication-dependent histone pre-mRNAs lack introns and undergo 3’-end processing that differs from cleavage coupled to polyadenylation. Histone pre-mRNAs contain two sequence elements essential for their 3’-end processing: a highly-conserved stem-loop structure and a purine-rich histone downstream element (HDE). Cleavage occurs between these two sequence elements and the polyadenylation step is omitted, giving rise to mature histone mRNAs that end with the stem-loop followed by a 4–5 nucleotide tail.

Biochemical studies of the 3’-end processing machinery that cleaves replication-dependent histone pre-mRNAs have shown that it is comprised of the stem-loop binding protein (SLBP), U7 small nuclear ribonucleoprotein (U7 snRNP), FLASH, and the histone pre-mRNA cleavage complex (HCC) [1, 3–6]. SLBP binds the 3’ stem-loop in the pre-mRNA and remains bound after mRNA maturation, and functions in translation [7, 8]. The 3’ stem-loop also recruits the 3’-5’ exoribonuclease 3’hExo [9, 10], which is not essential for processing [11] but trims the processed histone mRNAs and initiates degradation of histone mRNAs in the cytoplasm [12]. The core U7 snRNP consists of two integral and stably associated components: ~60-nucleotide U7 snRNA and a unique Sm ring, which contains Lsm10 and Lsm11 in place of the spliceosomal SmD1 and SmD2 [13, 14]. The U7 snRNP recognizes the pre-mRNA through base-pairing between the 5’-end of U7 snRNA and the HDE [15, 16]. SLBP bound to the upstream stem-loop stabilizes this interaction, likely by directly or indirectly contacting a subunit(s) of U7 snRNP [17].

**Fig 1:**
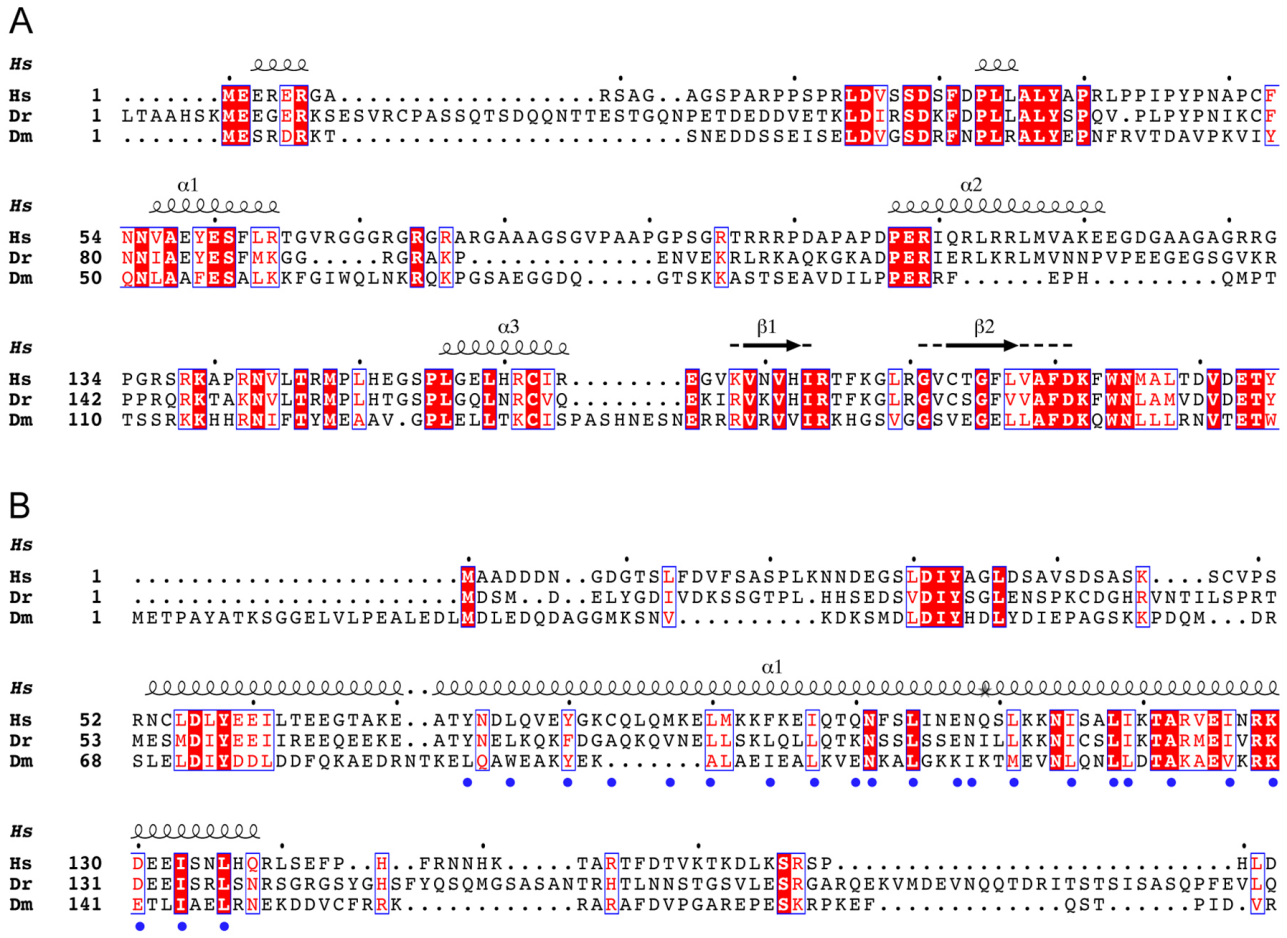
Multiple protein sequence alignment of FLASH and Lsm11 N-terminal domains. (A) Lsm11 N-terminal domain. (B) FLASH N-terminal domain. Alignment was carried out with Clustal Omega [60, 61] and the results displayed with ESPript [62]. Secondary structure for FLASH is based on the structure of human FLASH NTD, while that for Lsm11 is based on Psipred [63] secondary structure prediction of human Lsm11. Conserved residues are highlighted in red with white fonts, semi-conserved residues in red fonts, and other residues in black fonts. Blue dots indicate residues at the FLASH dimer interface. Gaps are indicated by dotted lines. Species abbreviations: Hs, *Homo sapiens* (human); Dr, *Danio rerio* (zebrafish); Dm, *Drosophila melanogaster* (fruit fly).

Lsm11 has an extended N-terminal domain (Fig 1A) that is unique among members of the functionally characterized Sm proteins. Through yeast two-hybrid and pull-down studies, this region was found to interact with the N-terminal region of FLASH (Fig 1B) [3]. FLASH, Flice-associated huge protein, was originally discovered as a protein involved in Fas-mediated apoptosis [18] and later in regulation of expression of several genes, including oncogenes [19, 20]. Subsequent studies showed that FLASH localizes to Histone Locus Bodies in the nucleus, suggesting a role in expression of histone genes [21], and that it is essential for histone pre-mRNA processing [3].

Biochemical studies revealed that the interacting N-terminal regions of Lsm11 and FLASH form a docking platform that recruits the HCC to the U7 snRNP [22]. The HCC is composed of a specific subset of proteins that also participate in cleavage and polyadenylation [23, 24], including the endonuclease CPSF-73, CPSF-100, symplekin and CstF-64 [1, 25–27]. Mutational studies on FLASH identified an LDLY motif (residues 55–58 in human FLASH, Fig 1B) as essential for binding the HCC, while residues 100–139 are involved in Lsm11 binding [22, 28].

The molecular details of how FLASH acts as a mediator between Lsm11 and HCC are still unclear. To shed some light on the essential role of FLASH in 3’-end processing of replication-dependent histone pre-mRNA processing [3], we carried out structural studies on the human FLASH N-terminal domain (NTD) encompassing residues 51–137 using X-ray crystallography. We also performed biophysical studies on the FLASH NTD and the FLASH NTD-Lsm11 NTD complex to characterize their oligomeric states and the stoichiometry of their complex.

## Results

### FLASH NTD forms a coiled-coil dimer

**Fig 2:**
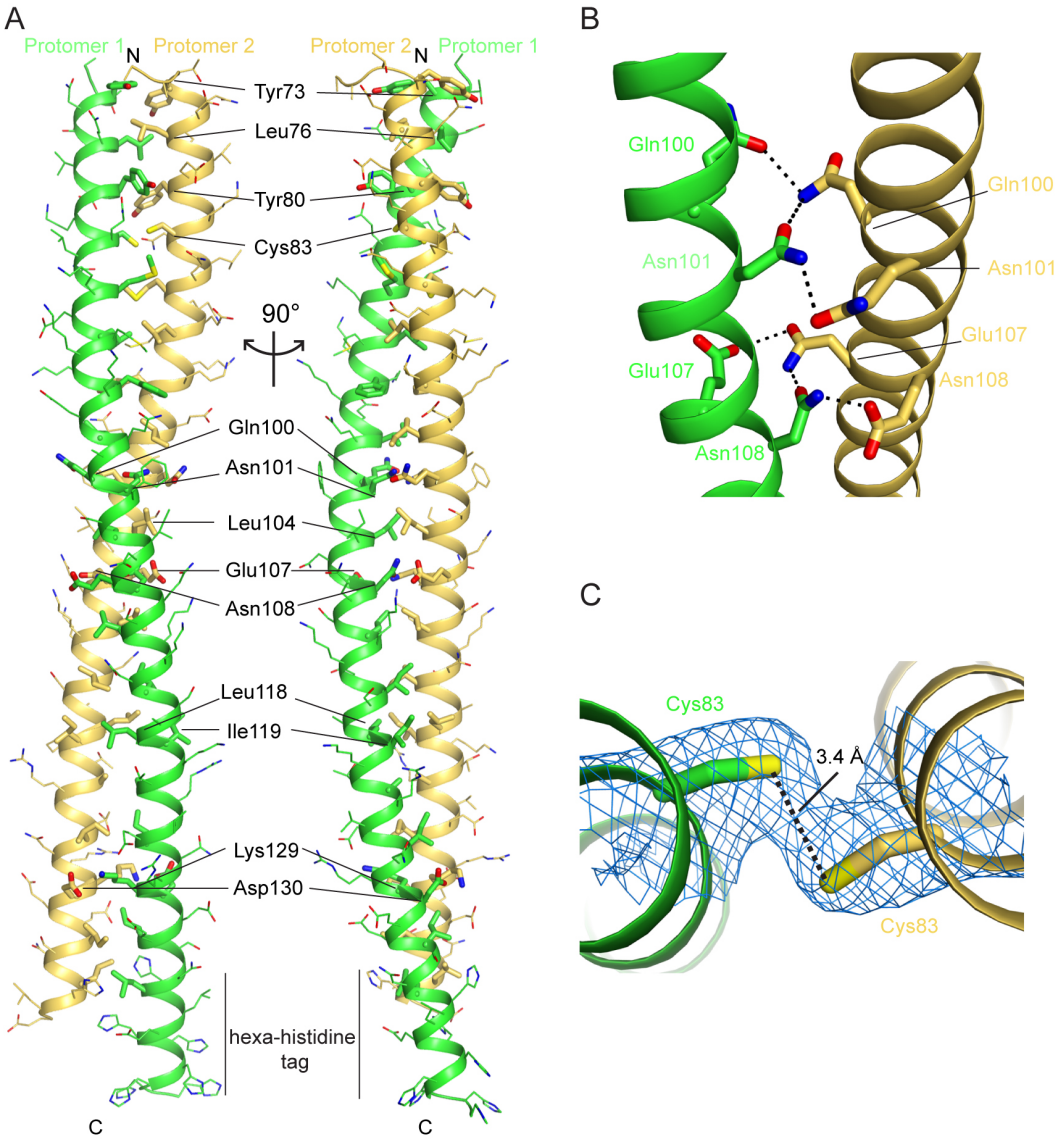
Structure of FLASH NTD dimer. (A) Two views of the FLASH NTD forming a coiled-coil dimer with respective protomers colored in green and yellow orange. The hexahistidine-tag was observed in protomer 1. Side chains of residues involved in the dimer interface are shown as sticks, while the other side chains are shown as thin sticks. Colors of atoms: red, oxygen; blue, nitrogen; yellow, sulfur/selenium. (B) Hydrophilic interactions (black dashes) formed by Gln100/Asn101 and Glu107/Asn108 respectively. (C) 2F_o_–F_c_ electron density for the Cys83 side chains, contoured at 1σ (blue).

We determined a structure of the wild-type human FLASH NTD at 2.6 Å resolution using X-ray crystallography (Table 1). The initial phases were obtained by the single anomalous dispersion (SAD) method using crystals of selenomethionyl FLASH NTD. The structure showed that FLASH NTD forms a coiled-coil dimer consisting of two parallel α-helices, one from each protomer (Fig 2A). However, only residues 71 to 137 were observed in this structure, even though the expression construct contained residues 51–137. Residues 51–70, which include the LDLY motif previously shown to be essential for histone pre-mRNA processing [28] and for binding the HCC [22], are disordered in this crystal. The first 30 residues of FLASH are poorly conserved among homologs (Fig 1B), although there is substantial conservation from *Drosophila* to mammals for residues 55–137 in the N-terminal segment.

**Table 1:** Summary of crystallographic information.

	SeMet FLASH NTD WT	FLASH NTD C54S/C83A mutant crystal form 1	FLASH NTD C54S/C83A mutant crystal form 2
**Data Collection**			
Space group	*C*2	*P*1	*C*2
Cell dimension			
a, b, c (Å)	66.8, 43.8, 65.0	39.3, 41.1, 61.0	63.5, 44.4, 68.4
α, β, ɣ (°)	90, 114.5, 90	95.6, 105.2, 113.4	90, 98.6, 90
Resolution (Å)	35.5–2.61 (2.77–2.61)	36.7–2.1 (2.18–2.1)	36.2–2.58 (2.74–2.58)
R_merge_ (%)	5.5 (49.3)	5.8 (42.8)	7.9 (46.7)
CC_1/2_	(0.846)	(0.822)	(0.898)
I/σI	14.2 (2.7)	18.2 (3.2)	13.4 (3.8)
Completeness (%)	99.3 (98.3)	97.9 (97.7)	99.6 (99.2)
Redundancy	3.8 (3.8)	3.9 (3.4)	3.8 (3.7)
**Refinement**			
Resolution (Å)	35.51–2.61 (2.87–2.61)	36.72–2.1 (2.21–2.1)	36.22–2.58 (2.84–2.58)
No. of reflections	10144 (2517)	18627 (2504)	11536 (2843)
*R*_work_/*R*_free_ (%)	0.199/0.252	0.211/0.254	0.214/0.249
No. of atoms			
Protein	1200	2378	1400
Ligand/ion		16	
Water	6	58	10
Average B-factors			
Protein	78.9	55.1	68.1
Ligand/Ion		81.5	
Water	73.1	61.9	55.3
R.m.s. deviations			
Bond length (Å)	0.004	0.003	0.003
Bond angles (°)	0.67	0.41	0.53
PDB entry code	6ANO	6AOZ	6AP0

Residues 71–137 observed in the structure form a single α-helix. The length of the coiled-coil FLASH NTD dimer is approximately 100 Å, excluding the C-terminal hexahistidine tag observed for one of the protomers. The protomers do not superimpose perfectly onto each other (r.m.s.d. ~1.5 Å), and one of them appears to adopt a straighter conformation (S1 Fig). For each protomer, the buried surface area is ~1800 Å^2^ (~25% of its total surface area, calculated using the program PISA [29]). The majority of the FLASH dimer interface residues are leucines and isoleucines, forming the bulk of the hydrophobic interactions (Fig 2A). The leucines and isoleucines are interspersed with other residues that form either polar or non-polar interactions. Other hydrophobic interactions are formed by bulky residues such as Tyr73, Tyr80, and Phe94, as well as Met87 (selenomethionine in this structure). Hydrophilic interactions include residues Gln100/Asn101 and Glu107/Asn108 near the mid-section of the structure (Fig 2B), and an ion pair between Lys129 and Asp130 at the C-terminal end of the coiled-coil (Fig 2A).

### FLASH NTD double cysteine mutant forms a similar dimer

We also observed that Cys83 is situated in the dimer interface with the thiol side chains from the two protomers positioned near one another (Fig 2C). While the electron density did not provide conclusive evidence for the existence of a disulfide bond, and the two sulfur atoms are separated by 3.4 Å distance in the current model, the structure raises the possibility that the observed FLASH NTD dimer might be mediated by a disulfide connection, which is unlikely to occur in the reducing environment in the nucleoplasm, the site of 3’-end processing.

**Fig 3:**
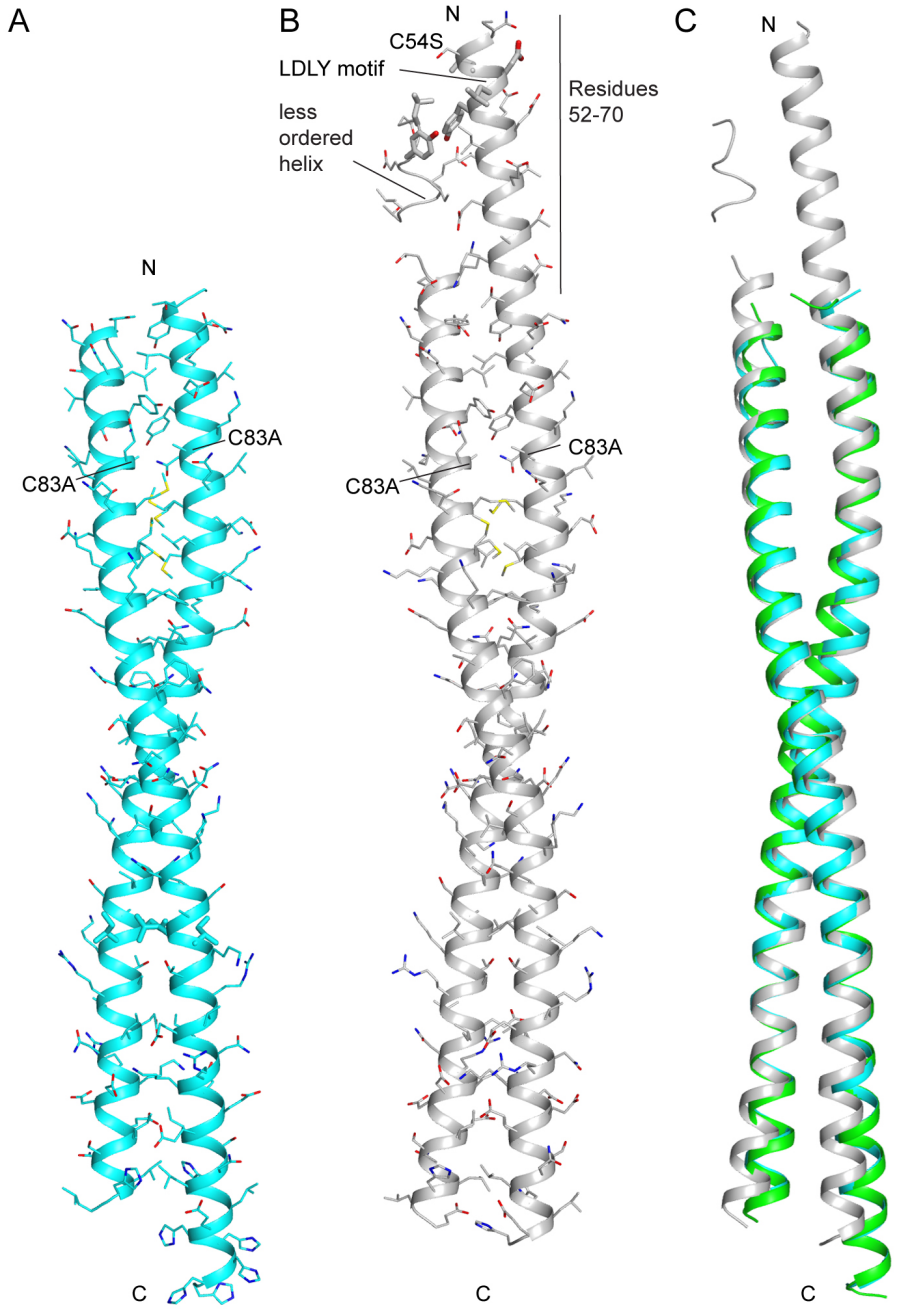
Structures of FLASH NTD C54S/C83A dimer. (A) Structure of FLASH NTD C54S/C83A crystal form 1 (resolution 2.1 Å) showing FLASH dimer without the presence of a disulfide bond. (B) Structure of FLASH NTD C54S/C83A crystal form 2 (resolution: 2.6 Å) showing observable residues 52–70 that adopt a helical structure on protomer 1, and are less ordered in protomer 2. The LDLY motif essential for binding the HCC is shown as sticks. (C) Superimposition of the structures of FLASH NTD C54S/C83A crystal forms 1 (cyan) and 2 (gray) with wild-type FLASH NTD dimers (green).

To rule out the possibility that the observed FLASH NTD dimer is a crystallographic artifact caused by oxidized cysteine residues, we determined the structures of the FLASH NTD C54S/C83A double mutant in two different crystal forms at 2.1 and 2.6 Å resolution, respectively (Table 1). In addition to Cys83, we also mutated the other Cys residue in the FLASH NTD, Cys54, in case it formed a disulfide as well even though the residue was disordered in the structure. The two structures adopt the same coiled-coil dimer (Fig 3A and 3B) as the wild-type FLASH NTD (Fig 3C), confirming that FLASH NTD dimer formation does not require the Cys83 disulfide. The individual protomers of these two mutant dimers also show differences, similar to those observed for the wild-type dimer (S1 Fig).

Interestingly, residues 53–70 from one of the protomers in the structure at 2.6 Å resolution are stabilized by crystal packing, showing strong electron density corresponding to an α-helix (Fig 3B). This protomer appears to form a single long α-helix from residues 53 to 137. The other protomer showed weak electron density for residues 56–62, while residues 51–55 and 63–66 are not observed (Fig 3B). These N-terminal residues are disordered in the other two structures (Fig 3C).

Overall, our structural data suggest that FLASH NTD alone forms a stable coiled-coil dimer from residues 71–137 while residues 53–70 can form another helix. It appears that the helix for residues 53–70 does not dimerize, and it may also be structurally independent of residues 71–137, even though a single long helix (residues 53–137) is observed in one crystal form. The LDLY motif (residues 55–58) essential for binding the HCC is situated in the N-terminal helix (Fig 3B). Whether the flexibility of this helix is a feature needed for binding the HCC will need to await further investigation.

### FLASH NTD mutations can affect Lsm11 NTD binding but not dimerization

**Fig 4:**
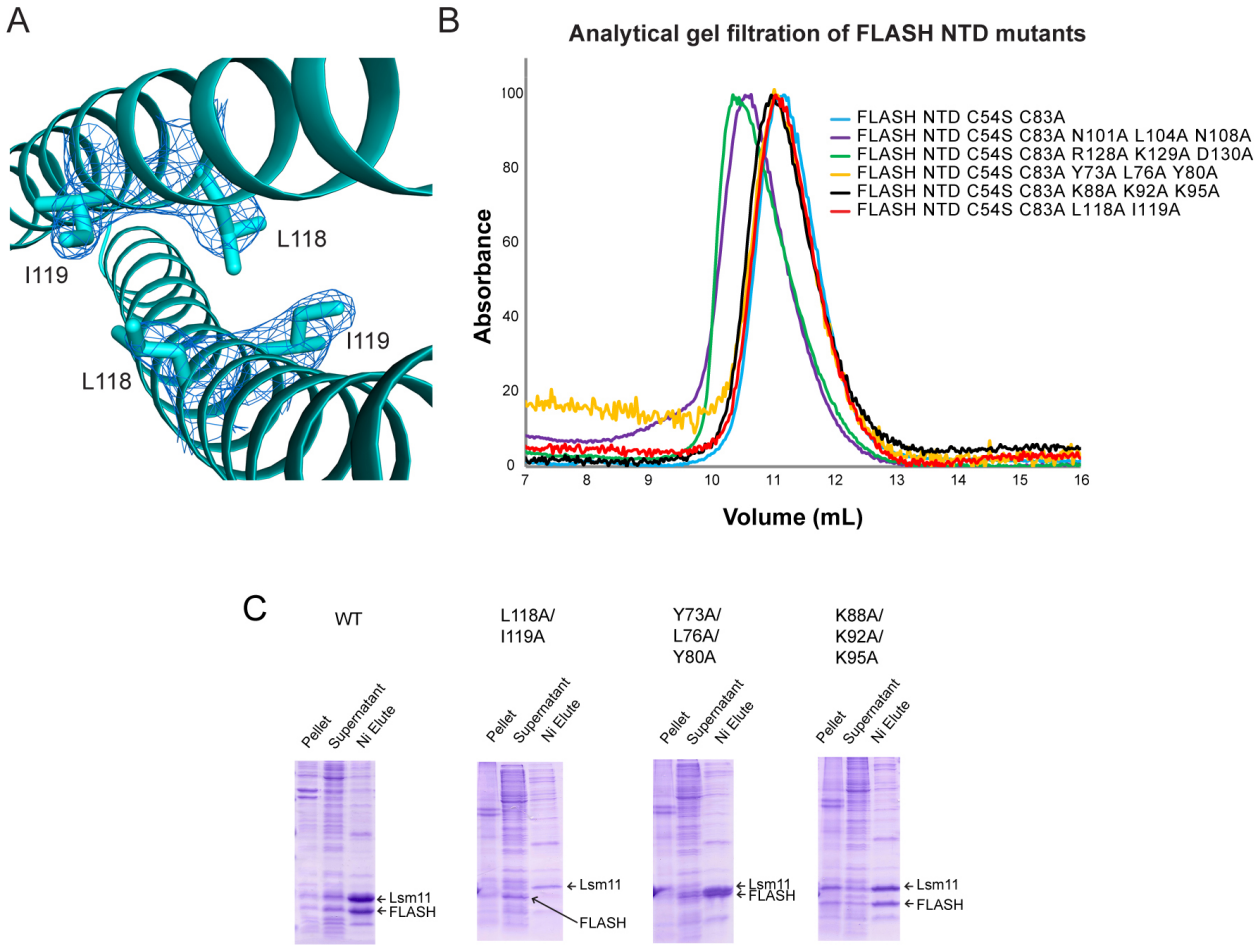
Biophysical analysis of FLASH NTD mutants. (A) Close-up view of Leu118 and Ile119 with 2F_o_–F_c_ electron density of the side chains (sticks) contoured at 1σ (blue). Coordinates and the electron density are derived from structure of FLASH NTD C54S/C83A crystal form 1 at 2.1 Å resolution. (B) Superose-12 analytical gel filtration profiles of FLASH NTD proteins. Peak heights are scaled to an arbitrary unit of 100. (C) SDS-PAGE analysis of co-purification of His-tagged Lsm11 NTD and wild-type and mutant FLASH NTD. All FLASH mutants have additional C54S/C83A mutations. Pellet and supernatant are the insoluble and soluble fractions, respectively, of the cell lysate.

The region of FLASH NTD that interacts with Lsm11 has been previously mapped to residues 100–137 [3, 13, 28]. Because our structural data indicated that this region forms a dimer, we investigated the role of FLASH NTD dimerization in Lsm11 binding. Previous pull-down studies showed that substituting Leu118 and Ile119 with alanines abolished the ability of FLASH to bind Lsm11 [28]. According to our FLASH NTD structures, both Leu118 and Ile119 are situated at the dimer interface (Fig 4A). It was possible that these mutations disrupt FLASH NTD dimerization, thereby affecting Lsm11 binding. To test this possibility, we generated the FLASH NTD L118A/I119A mutant in the background of C54S/C83A mutations and investigated its oligomeric state by analytical gel filtration (Fig 4B). Our results showed that this mutant had a similar migration behavior as the C54S/C83A mutant control, indicating that the deleterious effect of L118A/I119A mutation on binding Lsm11 is not due to the disruption of FLASH NTD dimerization.

We next investigated the oligomeric states of the mutants Y73A/L76A/Y80A and N101A/L104A/N108A, each containing substitutions of three consecutive residues in the dimer interface (Fig 2A). The first mutated cluster is located closer to the N-terminal end, while the second cluster is located near the middle of the NTD. In addition, since Lys129 and Asp130 form an ion pair in the dimer interface, we also replaced these two charged residues as well as the preceding Arg128 with alanines, generating the R128A/K129A/D130A mutant. Our gel filtration results showed that the Y73A/L76A/Y80A mutant had essentially the same migration behavior as the C54S/C83A control, while the N101A/L104A/N108A and R128A/K129A/D130A mutants actually migrated faster (Fig 4B). As a control, we made the K88A/K92A/K95A mutant, changing three residues located outside of the dimer interface. As expected, the migration behavior of this mutant was nearly the same as that for the C54S/C83A protein (Fig 4B). Our analytical ultracentrifugation studies on the N101A/L104A/N108A mutant suggested that it might be trimeric in solution (Table 2, see below), suggesting that the mutation has perturbed the structure of the NTD. Therefore, the N101A/L104A/N108A and R128A/K129A/D130A mutants will not be described further.

**Table 2:** Molecular weights for FLASH NTD, Lsm11 NTD and their complex from SEC-MALS experiments.

Sample	Calculated MW (kDa)	Observed Mass distribution (kDa)	Observed MW at peak apex (kDa)	Stokes radius (nm)	Oligomeric state
FLASH NTD alone	11.3	20.8–21.7 (high salt)	20.8 (high salt)	3.4	Homo-dimer
		19.0–23.5 (low salt)	21.7 (low salt)	3.5	Homo-dimer
FLASH NTD-Lsm11 NTD complex	FLASH NTD: 10.2 Lsm11 NTD: 13.6	26.0–34.4 (high salt)	30.6 (high salt)	3.9	2:1 complex (FLASH NTD dimer with Lsm11 NTD monomer, calculated MW 34.0 kDa)
		26.0–55.0 (low salt)	34.0 (low salt)	4.3	2:1 complex

While the mutations were not able to disrupt the FLASH NTD dimer, we tested whether they affect the interactions with Lsm11 NTD. For these experiments, we co-expressed His-tagged Lsm11 NTD with un-tagged FLASH NTD in *E*. *coli* and monitored whether FLASH NTD could be co-purified by the nickel-NTA agarose beads. The results showed that the Y73A/L76A/Y80A and K88A/K92A/K95A mutants still interacted with Lsm11 NTD, while the L118A/I119A mutant could no longer interact with Lsm11 NTD (Fig 4C), consistent with earlier data [28]. These experiments further demonstrated that the loss of binding between the FLASH NTD mutant and Lsm11 NTD is not linked to the dissociation of FLASH dimer.

### FLASH NTD-Lsm11 NTD complex is a 2:1 heterotrimer

**Fig 5:**
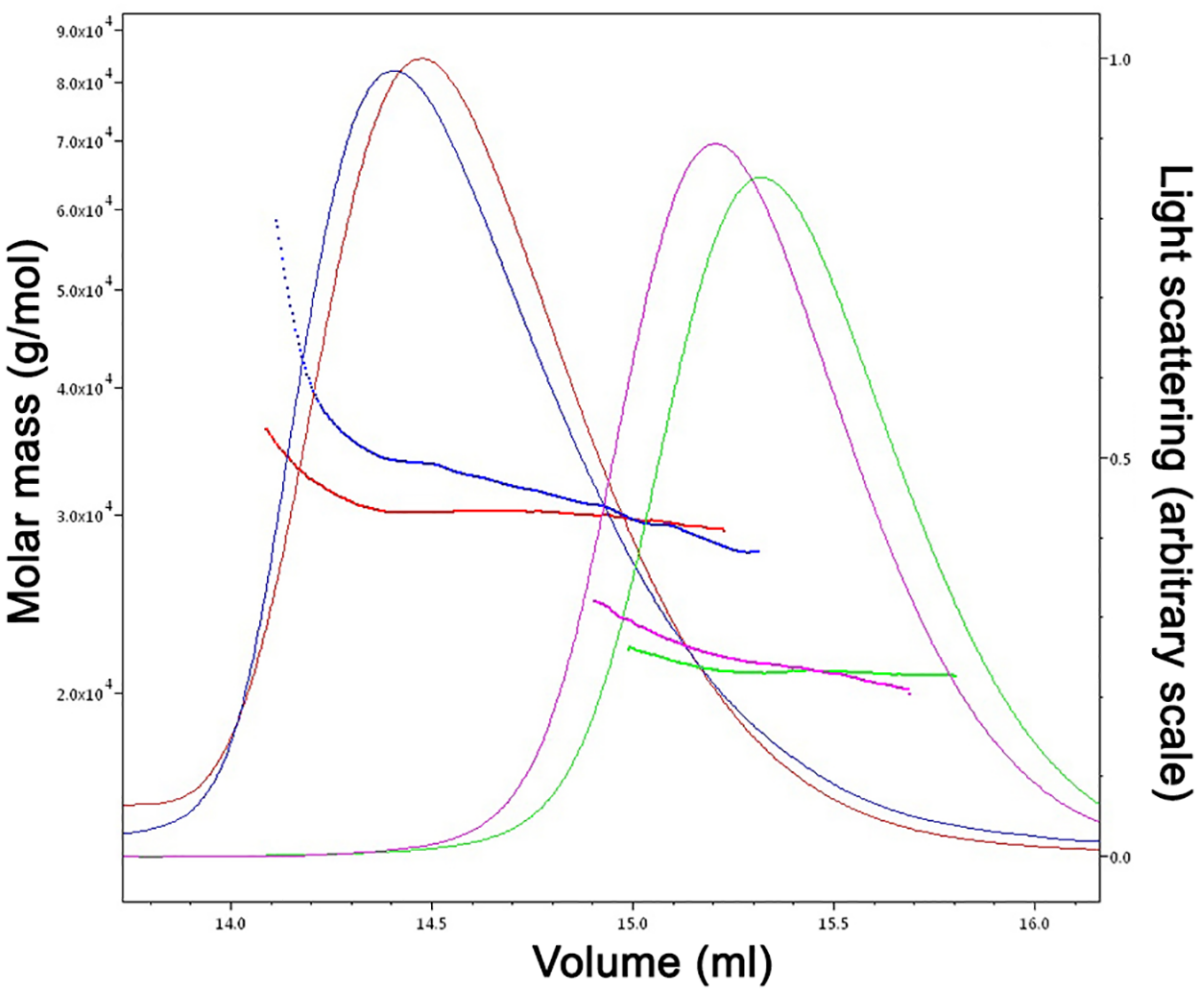
Size exclusion chromatograph-multi-angle light scattering of FLASH NTD-Lsm11 NTD complex and FLASH NTD C54S/C83A. SEC-MALS traces (with superimposed calculated molar mass traces) of FLASH NTD-Lsm11 NTD complex at 500 mM NaCl (blue), FLASH NTD C54S/C83A at 500 mM NaCl (purple), FLASH NTD-Lsm11 NTD complex at 250 mM NaCl (red), and FLASH NTD C54S/C83A at 250 mM NaCl (green). Left axis, molar mass; right axis, light scattering signal; bottom axis, elution volume.

Given the ability of the FLASH NTD to dimerize, we next characterized the stoichiometry of the FLASH-Lsm11 complex. We co-expressed FLASH NTD C54S/C83A double mutant and His-tagged Lsm11 NTD (residues 23–130) in *E*. *coli* and purified their complex, demonstrating a stable interaction between the two proteins (S2 Fig). Extensive efforts at producing diffraction quality crystals of the FLASH NTD-Lsm11 NTD complex have so far been unsuccessful. To obtain estimates for the molar masses of the FLASH NTD-Lsm11 NTD complex as well as the FLASH NTD C54S/C83A mutant alone, we performed size exclusion chromatography multi-angle light scattering (SEC-MALS) experiments using buffers with high (500 mM) and low (250 mM) NaCl concentrations (Fig 5, S3 Fig). At high-salt concentration, both the FLASH NTD-Lsm11 NTD complex and FLASH NTD alone eluted in single peaks. However, the FLASH NTD-Lsm11 NTD complex peak had a trailing edge, suggesting some dissociation of the complex during chromatography. The weight-averaged molar masses of the samples eluting in the peaks are 31 kDa for the FLASH NTD-Lsm11 NTD complex (with a Stokes radius of 3.9 nm) and 21 kDa for FLASH NTD C54S/C83A mutant alone (with a Stokes radius of 3.4 nm). The molar mass of the FLASH NTD-Lsm11 NTD complex decreased gradually from 34.4 kDa (leading edge of peak) to 26.0 kDa (trailing edge of peak). For FLASH NTD C54S/C83A, the molar mass decreased slightly from 21.7 kDa (leading edge) to 20.8 kDa (trailing edge), indicating that it formed a stable dimer.

In the low-salt buffer, the positions of the peaks for both the FLASH NTD-Lsm11 NTD complex and FLASH NTD C54S/C83A mutant were slightly shifted to the left, suggesting a more extended structure for both. A small amount of higher order structures was present for the FLASH NTD-Lsm11 NTD complex suggesting the formation of aggregates in low-salt buffer condition. As in the high-salt buffer, the FLASH NTD-Lsm11 NTD complex peak had a trailing edge. The weight-averaged molar masses of the samples eluting in the peaks are 34.3 kDa for FLASH NTD-Lsm11 NTD complex (with a Stokes radius of 4.3 nm) and 21.8 kDa for FLASH NTD C54S/C83A (with a Stokes radius of 3.5 nm). Due to the presence of higher order structures, the molar mass of the FLASH NTD-Lsm11 NTD complex decreased gradually from 55 kDa and higher (leading edge of peak) to 26 kDa (trailing edge of peak). The FLASH NTD C54S/C83A molar mass decreased from 23.5 kDa (leading edge) to 19 kDa (trailing edge). Overall, the polydispersity of the FLASH NTD-Lsm11 NTD complex was slightly higher in low-salt buffer.

Based on the calculated molecular weights for Lsm11 NTD and FLASH NTD, the results from SEC-MALS showed that the FLASH NTD-Lsm11 NTD complex is a heterotrimer consisting of 2 molecules (a dimer) of FLASH and 1 molecule of Lsm11, while FLASH NTD C54S/C83A is a dimer (Table 2).

### Analytical ultracentrifugation studies

**Fig 6:**
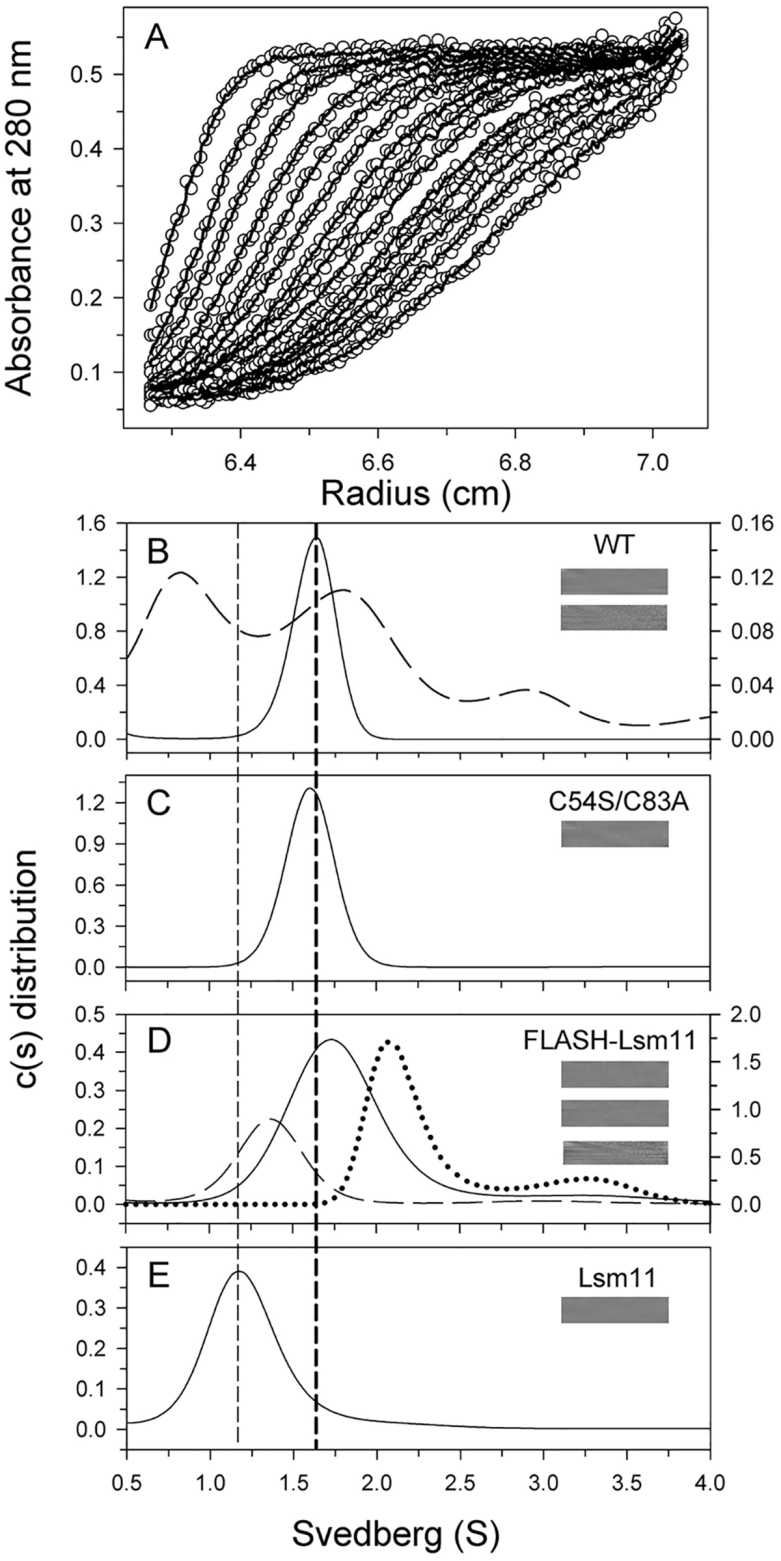
Analytical ultracentrifugation studies of FLASH NTD, FLASH NTD mutants, Lsm11, and FLASH NTD-Lsm11 NTD complex. (A) Typical traces of absorbance at 280 nm of the protein in 20 mM Tris (pH 7.5) buffer during the sedimentation velocity experiment. The protein concentration was 1 mg/ml. For clarity, only every fifth scan is shown. The symbols represent experimental data and the lines are the results obtained after being fitted to the Lamm equation using the SEDFIT program [57]. (B-E) Continuous c(s) distribution of FLASH NTD wild-type, C54S/C83A mutant, FLASH NTD-Lsm11 NTD complex and Lsm11 NTD. The distributions of the proteins at concentrations of 1 mg/ml (B-D) and 0.8 mg/ml (E) are shown by solid lines and those at concentrations of 0.1 mg/ml (B) and 0.05 mg/ml (D) are shown by dashed lines and that at 8 mg/ml (D) are showed by dotted line. The y-axis on the right is for the protein at a concentration of 0.1 mg/ml (B) and 8 mg/ml (D). The vertical dashed lines on the left and right indicate the monomer position of Lsm11 NTD and the dimer position of FLASH NTD, respectively. The residual bitmap of the raw data and the best-fit results are shown in the insets. The data are summarized in Table 2.

We also performed analytical ultracentrifugation (AUC) sedimentation velocity and sedimentation equilibrium experiments on the FLASH NTD-Lsm11 NTD complex, as well as on wild-type FLASH NTD, FLASH NTD C54S/C83A double mutant, and Lsm11 NTD alone as controls in the low-salt condition (Fig 6, S4 Fig). Our AUC results showed that wild-type FLASH NTD and the FLASH NTD C54S/C83A double mutant have similar sedimentation coefficients of 1.61 and 1.59 and frictional ratios of 1.81 and 1.86, consistent with them forming dimers in solution (Table 3). Both proteins have Stokes radius of 3.2–3.4 nm, while the long-axis radius is about 6.5 nm, based on the crystal structures. The *K*_d_ values for the wild-type and C54S/C83A NTD dimers are 0.18 and 0.05 μM, respectively (Table 3). Wild-type FLASH NTD showed serious aggregation below the concentration of 0.1 mg/ml, where some of the proteins apparently dissociated into a monomeric but more elongated form (sedimentation coefficients of 0.89, frictional ratio of 3.09 and Stokes radius of 5.5 nm). Lsm11 NTD alone showed a sedimentation coefficient of 1.22 with a frictional ratio of 1.81, suggesting that it exists as a monomer in solution.

**Table 3:** Summary of analytical ultracentrifugation data on FLASH NTD and Lsm11 NTD.

Protein	Concentration (mg/mL)	Sedimentation coefficient (s)^a^	Average frictional ratio^a^	M_r_ (kDa)^b^	R_s_ (nm)^b^	*K*_d_ (μM)
**FLASH NTD WT**	1	1.61	1.81	23.4	3.3	0.18 ± 0.004^c^
	0.3	1.56	1.79	21.7	3.2	
	0.1	0.89, 1.77	3.09	20.9, 62.9	5.5, 8.3	
**FLASH NTD C54S/C83A**	1	1.59	1.86	23.7	3.4	0.05 ± 0.002^c^
	0.3	1.56	1.79	21.8	3.2	
	0.08	1.64	2.01	28.5	4.0	
**FLASH NTD C54S/C83A - Lsm11 NTD**	8	2.1 (2.08^d^) 3.2 (3.03^d^)	1.64	25.4^d^ 49.7^d^	2.6 3.4	2.37 ± 0.02^e^
	1	1.75	1.72	24.9	3.3	
	0.2	1.37	1.78	17.8	3.0	
	0.05	1.36	1.99	20.7	3.5	
**Lsm11 NTD**	0.8	1.22	1.81	15.9	3.0	-
	0.2	1.26	1.68	15.3	2.8	
	0.05	1.33	1.76	21.4	3.7	
**FLASH NTD C54S/C83A/N101A/L104A/N108A**	1	1.63	2.46	37.4	5.3	-
	0.2	1.60	2.46	35.9	5.2	
	0.05	1.71	2.40	39.8	5.4	

^a^ The values were derived from fitting the AUC data to a continuous c(s) distribution model using the SEDFIT program [57].

^b^ The Stokes radii (R_S_) were calculated by the Sednterp program, based on the sedimentation coefficient and molecular weight (M_r_) from c(s) distribution analysis.

^c^ The values were derived from a global fit of the AUC data to a monomer-dimer self-association model by SEDPHAT [58].

^d^ The values were derived from fitting the sedimentation velocity and sedimentation equilibrium data to a global two discrete species model using the SEDPHAT [58].

^e^ The value was derived from a global fit of the AUC data to a A + B <—> AB hetero-association model by SEDPHAT [58].

The FLASH NTD-Lsm11 NTD complex showed a shift in its sedimentation coefficient value from 1.36 to 3.2 with increasing concentration of the complex (Table 3, Fig 6D). This observation indicates that the dissociation and association of the complex is a rapid event [30] and the exact molar mass of the complex cannot be accurately estimated. The broad range of observed molecular weight by AUC (17.8 to 49.7 kDa, Table 3) is similar to that seen in the SEC-MALS experiment. Nevertheless, we were able to obtain a *K*_d_ value of 2.4 μM for the complex (Table 3).

#### Crosslinking studies

We used glutaraldehyde to crosslink the FLASH NTD-Lsm11 NTD complex, FLASH NTD C54S/C83A, and FLASH NTD and analyzed it on SDS-PAGE (S5 Fig). We observed the strong presence of dimers for both FLASH wild-type and C54S/C83A double mutant and weaker presence of higher oligomers (trimer, tetramer etc.). The higher oligomers became less apparent at lower concentration (0.01 mg/mL) of the protein, suggesting that they are probably due to random collisions of monomer/dimer in solution. Dimer and trimer species were also observed for the FLASH NTD-Lsm11 NTD complex but Lsm11 NTD did not appear to be substantially crosslinked to FLASH or to itself, possibly due to the fact that it has only one lysine residue. Therefore, we conclude that the dimer and trimer species for the complex were probably crosslinked FLASH, as observed for the FLASH alone samples, and the cross-linking experiments by themselves did not provide conclusive information about the stoichiometry of FLASH NTD-Lsm11 NTD complex.

## Discussion

Human FLASH is a protein of 220 kDa that has been implicated in a broad spectrum of cellular processes. In spite of these diverse and important functions, the structural organization of FLASH remains largely unknown. Although FLASH consists of nearly 2,000 amino acid residues, the functions of only three small regions of the protein are understood: a 100 residue segment in the N-terminus required for histone pre-mRNA processing, the C-terminal segment which forms a SANT/Myb-like domain, interacts with the C-terminal region of NPAT, and is required for localization to the histone locus body [31]; and a small central region which binds Ars2 [32] is essential for cell cycle progression.

Our crystallographic studies of FLASH NTD demonstrate that residues 71–137 adopt a continuous and stable α-helical fold and mediate the formation of a coiled-coil dimer between two FLASH molecules. This α-helical fold might also extend to residues 53–70, encompassing the LDLY motif, but this region is unlikely to contribute to the dimerization interface. Our data are consistent with recent H/D exchange studies [33], which showed that residues 75–136 underwent slow H/D exchange, indicative of extensive secondary structure in this region. Residues 58–62 exchanged significantly faster than the 75–136 region but slower than the directly surrounding sequences, suggesting the presence of a more dynamic secondary structure in the vicinity of the LDLY motif [33].

That amino acids in the N-terminal region of FLASH may fold into a coiled-coil domain was first predicted by bioinformatics [34]. In addition, biochemical studies demonstrated that ectopically-expressed FLASH can self-associate in tissue culture cells and that this self-association requires the N-terminal 200 residues [32]. These data, in conjunction with our current crystallographic study, strongly support the notion that the N-terminal domain of FLASH exists in solution as a coiled-coil dimer. We changed up to three consecutive residues in the dimer interface but failed to convert FLASH into monomers. The dimer interface of the FLASH NTD is extensive and local structural disturbances, such as the three consecutive residues that we mutated, are insufficient to prevent dimerization. Interestingly, the L118A/I119A mutation in the interface of the coiled-coil dimer failed to disrupt FLASH dimerization but was sufficient to abolish the ability of FLASH to interact with Lsm11.

The N-terminal α-helical region that mediates FLASH dimerization overlaps substantially with the core Lsm11 binding site in FLASH mapped to amino acids 100–140, prompting the hypothesis that Lsm11 may interact with a FLASH dimer. SEC-MALS experiments on the complex provided strong evidence that the FLASH NTD-Lsm11 NTD complex is a 2:1 heterotrimer. While our AUC data confirmed that FLASH is a dimer, the stoichiometry of FLASH and Lsm11 in the FLASH NTD-Lsm11 NTD complex was less clear, likely due to dissociation of Lsm11 NTD from the FLASH NTD dimer during prolonged ultracentrifugation. Some dissociation of the complex was observed during the short time scale of the SEC-MALS experiment. That Lsm11 interacts with a FLASH dimer is also consistent with the data from H/D exchange experiments. While the region between amino acids 100–120 showed the slowest H/D exchange within the entire FLASH NTD (which we show here can form a dimer), this region underwent slower H/D exchange in the presence of Lsm11 and the reduced rate of exchange extended to amino acid 130 in FLASH [33]. Since H/D exchange occurs when hydrogen bonds are temporarily destabilized, this region of FLASH (residues 100–130) is in a more stable structure in the heterotrimer than in the homodimer.

Additional studies are required to determine the structure of the FLASH-Lsm11 heterotrimer and identify potential mechanisms that may regulate the binding of a FLASH dimer to Lsm11 to form the FLASH-Lsm11 heterotrimer. In animal cells, components of the transcription and 3’-end processing machinery are localized in Histone Locus Bodies (HLBs), nuclear domains that assemble at histone gene loci and are present throughout the cell cycle. Strikingly, histone gene expression is repressed during G1 phase and becomes activated only with the onset of S phase and DNA replication in response to cell cycle signals, including cyclin E/CDK2-mediated phosphorylation of NPAT, a universal coactivator of histone gene expression [35–39]. A growing body of evidence suggests that FLASH is targeted to HLBs as a separate entity rather than a subunit of the U7 snRNP. For example, mutations in either Lsm11 or FLASH that disrupt binding between their N-terminal domains do not affect localization of either FLASH or U7 snRNP to the *Drosophila* HLB, but abolishes processing *in vivo* [40, 41].

Our findings suggest that N-terminus of FLASH may be present as a homodimer throughout the cell cycle. In recent studies [33], we have found that a second region of Lsm11 interacts with the C-terminal region of FLASH, the same region of FLASH that binds to NPAT [31]. This interaction strengthens the overall binding between FLASH and Lsm11 and could be part of an extensive reorganization of the factors in the HLB to activate histone gene expression as a result of phosphorylation of NPAT by cyclin E/CDK2.

## Materials and methods

### Protein expression and purification of FLASH NTD

C-terminally hexahistidine-tagged FLASH NTD (residues 51–137) and FLASH NTD C54S/C93A mutant constructs were cloned into pET26b vector and over-expressed in *Escherichia coli* BL21 Star (DE3) strains (Novagen). The cells were induced using 0.4 mM isopropyl β-D-1-thiogalactopyranoside and grown for 18 h at 20°C. The cells were harvested by centrifugation and the pellets were re-suspended in lysis buffer (20 mM Tris (pH 7.5), 500 mM NaCl, 10 mM imidazole, 5% (v/v) glycerol, 17.8 μg/mL phenylmethane sulfonyl fluoride (PMSF) and 10 mM β-mercaptoethanol) and lysed by sonication. Cell lysates were then centrifuged at 25,000 x g for 40 min at 4°C. The supernatant was incubated with nickel beads for 1 h before being loaded onto a gravity flow column (Bio-Rad). The nickel beads were washed with buffer containing 20 mM Tris (pH 7.5), 500 mM NaCl, 40 mM imidazole, and 10 mM β-mercaptoethanol. The proteins were eluted with 20 mM Tris (pH 7.5), 500 mM NaCl, 500 mM imidazole and 10 mM β-mercaptoethanol. The eluted proteins were further purified using size-exclusion chromatography (Sephacryl S-300; GE Healthcare) with a buffer containing 20 mM Tris (pH 8.5), 250 mM NaCl, and 5 mM dithiothreitol (DTT). Relevant fractions from size-exclusion chromatography were pooled and the proteins were concentrated to 9.4 mg/mL (wild-type) and 11 mg/mL (mutant), and stored at -80°C.

The selenomethionyl FLASH NTD protein was prepared using the *Escherichia coli* B834 methionine-auxotroph strain grown in the LeMaster media supplemented with selenomethionine [42]. The protein was purified using the same protocol as the native protein, concentrated to 10 mg/mL and stored at –80°C.

### Protein crystallization

Selenomethionyl FLASH NTD and native FLASH NTD C54S/C83A mutant were crystallized in a sitting drop by vapor diffusion. The sitting drops were set up by mixing 1 μL of 4 mg/mL selenomethionyl FLASH NTD or 5 mg/mL FLASH NTD C54S/C83A mutant protein with 1 μL of well solution. The well solution for selenomethionyl FLASH NTD crystals contained 100 mM Tris (pH 8.0) and 18% (w/v) PEG 4000; for FLASH NTD C54S/C83A crystal form 1, 4% (w/v) tacsimate pH 7.0, 11% (w/v) PEG 3350; and for FLASH NTD C54S/C83A crystal form 2, 100 mM sodium formate, 15% (w/v) PEG 3350, 3% (v/v) 1,6-hexanediol. The crystals were harvested and soaked in mother liquor supplemented with 15% (v/v) (selenomethionyl FLASH NTD) or 20% (v/v) (FLASH NTD C54S/C83A) ethylene glycol as cryoprotectant before being flash frozen in liquid nitrogen.

### Data collection and structure determination

Initial X-ray diffraction data for selenomethionyl FLASH NTD were collected at APS beamline 24 ID-C with wavelength 0.9792 Å. Three datasets from three different crystals were processed using XDS and merged with XSCALE [43, 44]. The structure was solved by SAD using ShelxCDE [45] and the model built manually with the program Coot [46]. The final structure was then refined using a higher resolution dataset (2.6 Å) collected at ALS beamline 501 (wavelength: 0.9774 Å) and processed with XDS. Data for the structures of FLASH NTD C54S/C83A were collected using single crystals at APS beamline 24-ID-E (0.9792 Å wavelength for both). The datasets were processed using HKL2000 [47] (FLASH C54S/C83A crystal form 1) and XDS (FLASH C54S/C83A crystal form 2). Both structures were solved by molecular replacement using Phaser [48] with the selenomethionyl FLASH NTD structure as the search model. All three structures were refined using Phenix [49].

### Expression and purification of FLASH NTD-Lsm11 NTD complex and Lsm11 NTD

N-terminally hexahistidine-tagged Lsm11 NTD (residues 23–130) construct was cloned into pET28a vector. FLASH NTD C54S/C83A mutant construct (without tag) was cloned into MCS2 of pCDF Duet vector. Both plasmids were co-transformed into *E*. *coli* BL21 (DE3) Star and the genes were co-expressed. The proteins were purified using the same protocol as for the FLASH NTD proteins with the exception of using 20 mM Tris (pH 7.5), 500 mM NaCl, and 5 mM DTT as the size exclusion chromatography buffer. Relevant fractions corresponding to Lsm11 NTD-FLASH NTD C54S/C83A complex and excess Lsm11 NTD alone were pooled separately and concentrated to 5.3 mg/mL and 2.4 mg/mL respectively.

### Generation of mutant FLASH NTD constructs

Mutant FLASH NTD constructs were generated using site-directed mutagenesis PCR. Primers (see S1 Table) designed to mutate designated residues were used in PCR reactions to amplify plasmid templates encoding for wild type or mutant FLASH NTD (see S1 Table for templates used). 25 cycles of thermal cycling (98°C for melting, 55°C for annealing, 72°C for elongation) were performed using Phusion polymerase. PCR products were then digested with DpnI for 1 h at 37°C before being transformed into *E*. *coli* DH5α. Mutant constructs were confirmed by DNA sequencing.

### Analytical gel filtration of FLASH NTD mutants

The C-terminally hexahistidine-tagged FLASH NTD mutant constructs were cloned into pET26b vector and expressed in *E*. *coli* BL21 Star (DE3) cells. The mutant proteins were purified by nickel affinity column as detailed in the earlier section. The eluted protein from nickel affinity purification was then injected into Superose 12 analytical gel filtration column, pre-equilibrated with 20 mM Tris (pH 7.5), 250 mM NaCl, and 5 mM DTT. Analytical gel filtration was performed with a flow rate of 0.5 mL/min using 20 mM Tris (pH 7.5), 250 mM NaCl and 5 mM DTT as buffer.

### Co-purification of FLASH NTD mutants with His-tagged Lsm11 NTD

N-terminally hexahistidine-tagged Lsm11 NTD (residues 23–130) construct was cloned into pET28a vector. FLASH NTD mutant constructs (without tag) were cloned into MCS2 of pCDFDuet vector. Both plasmids were co-transformed into *E*. *coli* BL21 Star (DE3) and the genes were co-expressed in 5 mL LB media. The expressed proteins were co-purified with 15 μL of Ni-NTA agarose beads using the same buffers that were used for large-scale purification of FLASH NTD-Lsm11 NTD complex, and analyzed using SDS-PAGE.

### Analytical ultracentrifugation

The AUC experiments were performed on a XL-A analytical ultracentrifuge (Beckman Coulter) using an An-50 Ti rotor [50–56]. The sedimentation velocity experiments were performed using a double-sector *epon* charcoal-filled centerpiece at 20°C with a rotor speed of 42,000 rpm. Protein solutions of 0.05 to 1 mg/ml (330 μl) in a buffer containing 20 mM Tris (pH 8.5), 250 mM NaCl, and 5 mM DTT (low-salt condition) and reference (370 μl) solutions were loaded into the centerpiece, respectively. The absorbance at 280 nm was monitored in a continuous model with a time interval of 300 s and a step size of 0.003 cm. Multiple scans at different time intervals were then fitted to a continuous c(s) distribution model using the SEDFIT program [57]. Additionally, the results with the various different protein concentrations were globally fitted to monomer-dimer self-association or A + B <—> AB hetero-association model using the SEDPHAT program to calculate the dissociation constant (*K*_d_) [58].

To determine the precise molecular weight of the protein, the sedimentation equilibrium experiment was performed [59]. Three different samples (0.10–0.12 ml) were loaded into the sample channels of six-channel *epon* charcoal-filled centerpieces, and 0.11–0.13 ml buffers were loaded into the reference channels. The cells were then loaded into the rotor and run at speed of 10,000, 15,000, and 25,000 rpm each for 12 h at 20°C. Ten A_280_ nm scans with time interval of 8–10 min were measured for every different rotor speed to check the status of sedimentation equilibrium. Global analyses of combined sedimentation equilibrium and sedimentation velocity data were conducted with SEDPHAT using species analysis model [55].

### Size exclusion chromatography multi-angle light scattering (SEC-MALS)

FLASH NTD C54S/C83A-Lsm11 NTD complex and FLASH NTD C54S/C83A were loaded sequentially onto a Superdex 200 size exclusion column (24 mL) pre-equilibrated with 20 mM Tris pH 7.5, 500 mM NaCl, 5 mM DTT (high salt buffer) or 20 mM Tris pH 7.5, 250 mM NaCl, and 5 mM DTT (low salt buffer). The eluted samples first passed through a Wyatt multi-angle light scattering system (DAWN HELEOS-II) and then a Wyatt Trex refractometer. The data were analyzed using ASTRA version 6 software (Wyatt Technology, Santa Barbara, CA). The monomer peak of 3 mg/ml BSA was used for normalization, delay time determination, and band broadening correction using ASTRA.

### Glutaraldehyde crosslinking assay

Cross-linking reactions were carried out in 20 mM HEPES (pH 7.5), 500 mM NaCl. A final concentration of 0.1% (w/v) of glutaraldehyde was added to 0.1 mg/mL (total volume ~100 μL) and 0.01 mg/mL (total volume ~ 1 mL) of FLASH NTD C54S/C83A-Lsm11 NTD complex, FLASH NTD C54S/C83A, and FLASH NTD wildtype. Controls with protein concentrations of 0.1 mg/mL without glutaraldehyde were set up for each sample type. All samples were incubated at 37°C for 3 min then chilled on ice. A final concentration of about 100 mM of Tris pH 8.0 was added into each sample to quench the cross-linking reaction. All samples were concentrated to a volume ~20 μL using Sartorius Vivaspin^®^ 500 centrifugal concentrators with a molecular weight cut off of 10 kDa and finally analyzed by SDS-PAGE.

## Supporting information

S1 Fig Superimposition of all protomers from wildtype FLASH NTD (yellow orange and orange), FLASH C54S/C83A crystal form 1 (Mutant 1; cyan and teal), and FLASH C54S/C83A crystal form 2 (Mutant 2; gray and black). Protomers were superimposed using residues 71–100 from wildtype FLASH NTD protomer 1 as reference coordinates.
(TIF)

S2 Fig Sephacryl-300 (S-300) and SDS-PAGE analysis of purification of the Lsm11 NTD/FLASH C54S/C83A NTD complex. A) Sephacryl-300 gel filtration profile shows two peaks: peak 1 corresponds to the Lsm11 NTD/FLASH NTD C54S/C83A complex, peak 2 corresponds to excess Lsm11 NTD. Lsm11 contains the N-terminal hexa-histidine tag. B) SDS-PAGE analysis of nickel affinity eluate (labeled Ni/E), and corresponding fractions from S-300 chromatography. A molecular weight marker is situated on the far-left lane.
(TIF)

S3 Fig Additional data from SEC-MALS experiments. Light scattering (solid), refractive index (dotted), and MW information for (A) FLASH NTD in high salt buffer; (B) FLASH NTD in low salt buffer; (C) FLASH NTD-Lsm11 NTD in high salt buffer; and (D) FLAST NTD-Lsm11 NTD in low salt buffer.
(TIF)

S4 Fig Global analysis of FLASH-Lsm11 proteins at 8 mg/ml by AUC. The speed of centrifugation for sedimentation equilibrium experiment (A) was 10,000 rpm (squares), 15,000 rpm (circles), and 25,000 rpm (triangles) at 20°C each for 14 h. The velocity experiment (B) was 42,000 rpm (circles) at 20°C for 6 h. The solid lines in two panels are the best fit results from global analysis of the two discrete species models by SEDPHAT (57). The residuals of each fit are shown below the panels. The calculated sedimentation coefficients and M_r_ from the best fit results are shown in Table 2.
(TIF)

S5 Fig SDS-PAGE analysis of glutaraldehyde cross-linking of FLASH NTD C54S/C83A-Lsm11 NTD, FLASH NTD C54S/C83A, and FLASH NTD wild-type. In the FLASH NTD-Lsm11 NTD complex, FLASH NTD is the lower band (as it lacks a His tag compared to FLASH NTD alone), and Lsm11 NTD is the upper band. While the FLASH NTD band disappeared in the presence of glutaraldehyde, the Lsm11 NTD band mostly stayed the same. Therefore, probably very small amount of Lsm11 NTD (if any) got crosslinked in the reaction, consistent with the fact that it has only 1 Lys residue.
(TIF)

S1 Table Primers used for cloning and mutagenesis. (DOCX)
