# Supplementary figures and images for: The N-terminal domains of FLASH and Lsm11 form a 2:1 heterotrimer for histone pre-mRNA 3’-end processing
Source: PLoS One. 2017 Oct 11;12(10):e0186034. doi: 10.1371/journal.pone.0186034 (PMC5636114; doi:10.1371/journal.pone.0186034)

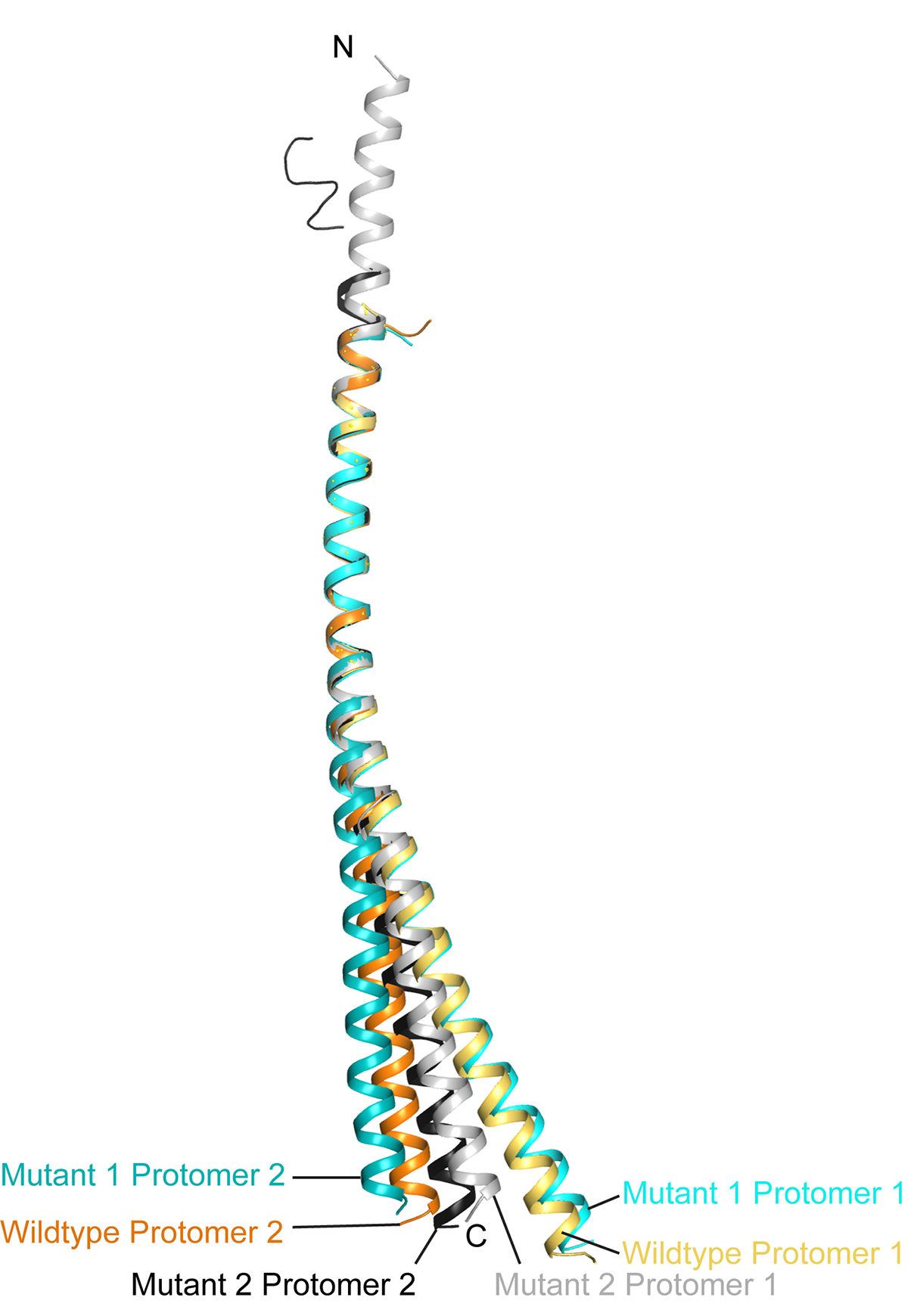

Supplement: S1 Fig — Protomers were superimposed using residues 71–100 from wildtype FLASH NTD protomer 1 as reference coordinates. (TIF) [file pone.0186034.s001.tif]

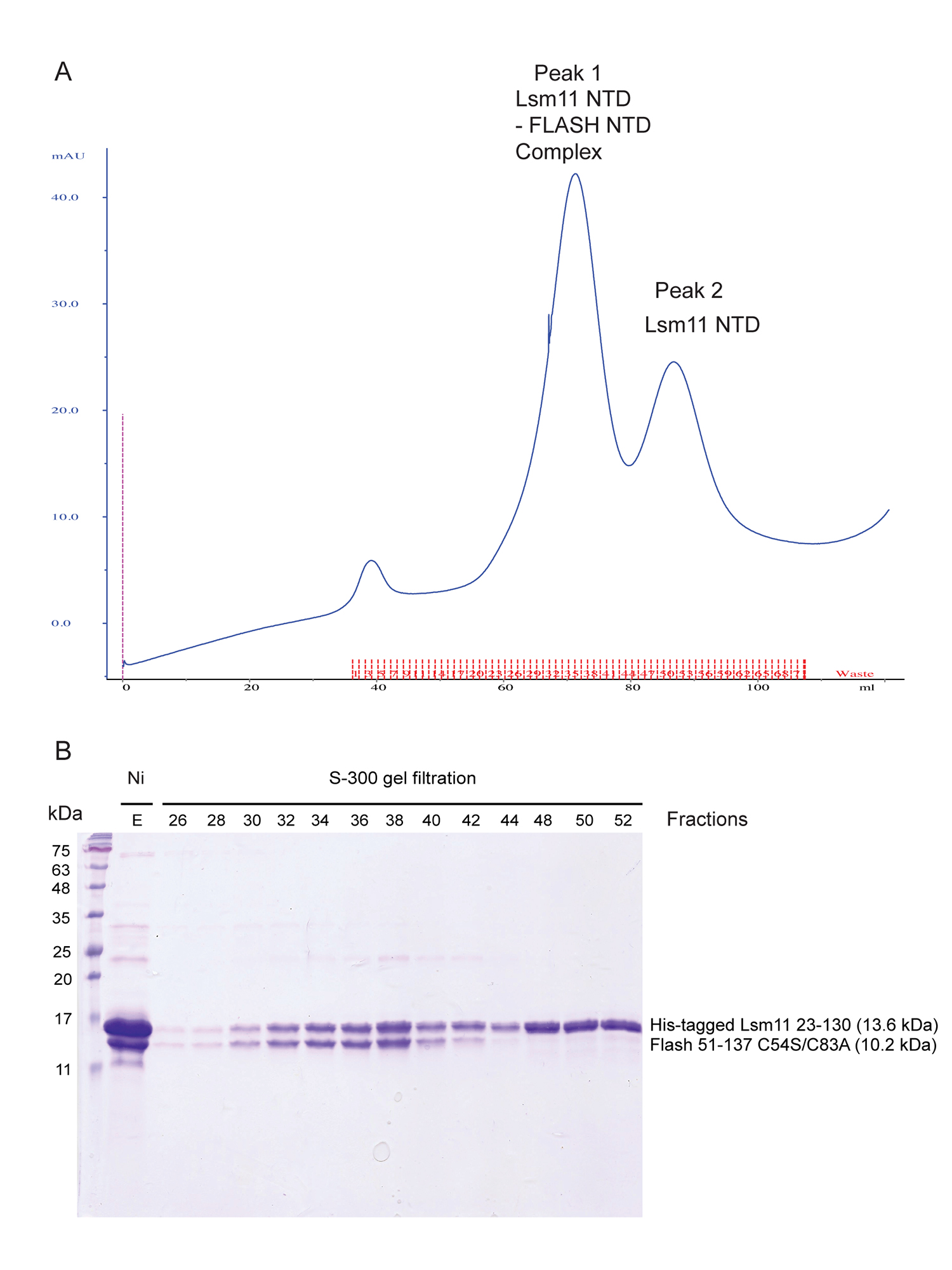

Supplement: S2 Fig — A) Sephacryl-300 gel filtration profile shows two peaks: peak 1 corresponds to the Lsm11 NTD/FLASH NTD C54S/C83A complex, peak 2 corresponds to excess Lsm11 NTD. Lsm11 contains the N-terminal hexa-histidine tag. B) SDS-PAGE analysis of nickel affinity eluate (labeled Ni/E), and corresponding fractions from S-300 chromatography. A molecular weight marker is situated on the far-left lane. (TIF) [file pone.0186034.s002.tif]

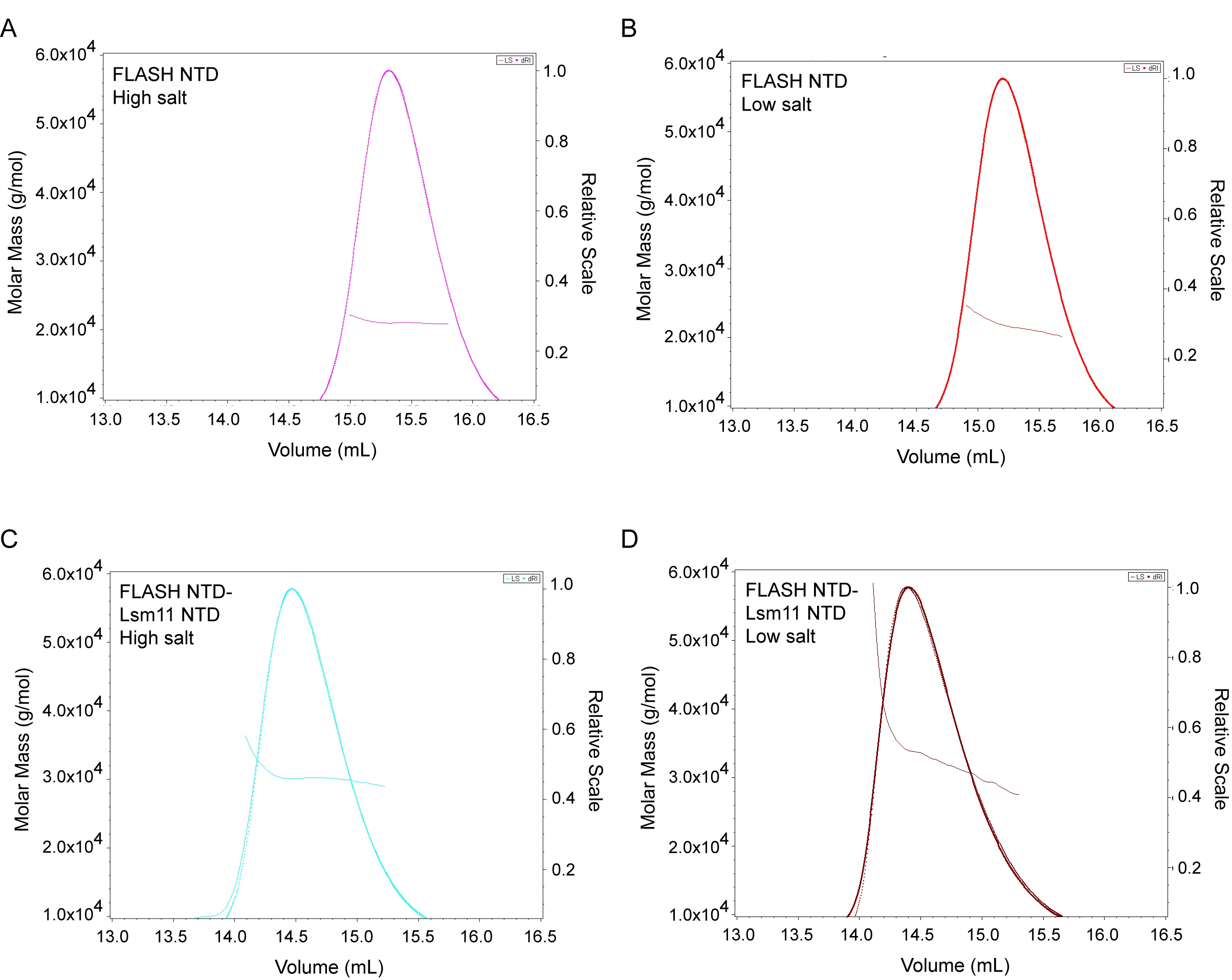

Supplement: S3 Fig — Light scattering (solid), refractive index (dotted), and MW information for (A) FLASH NTD in high salt buffer; (B) FLASH NTD in low salt buffer; (C) FLASH NTD-Lsm11 NTD in high salt buffer; and (D) FLAST NTD-Lsm11 NTD in low salt buffer. (TIF) [file pone.0186034.s003.tif]

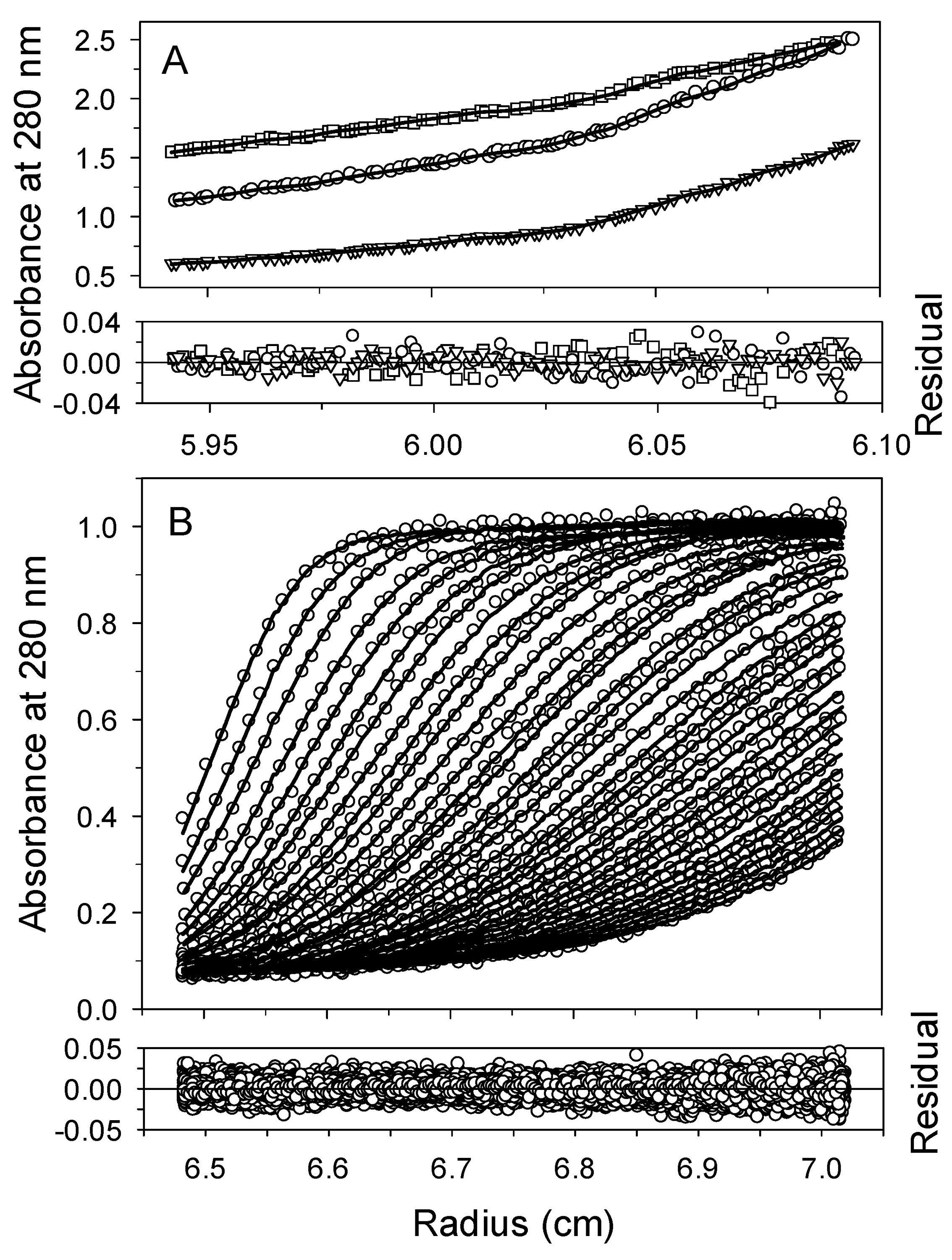

Supplement: S4 Fig — The speed of centrifugation for sedimentation equilibrium experiment (A) was 10,000 rpm (squares), 15,000 rpm (circles), and 25,000 rpm (triangles) at 20°C each for 14 h. The velocity experiment (B) was 42,000 rpm (circles) at 20°C for 6 h. The solid lines in two panels are the best fit results from global analysis of the two discrete species models by SEDPHAT (57). The residuals of each fit are shown below the panels. The calculated sedimentation coefficients and Mr from the best fit results are shown in Table 2. (TIF) [file pone.0186034.s004.tif]

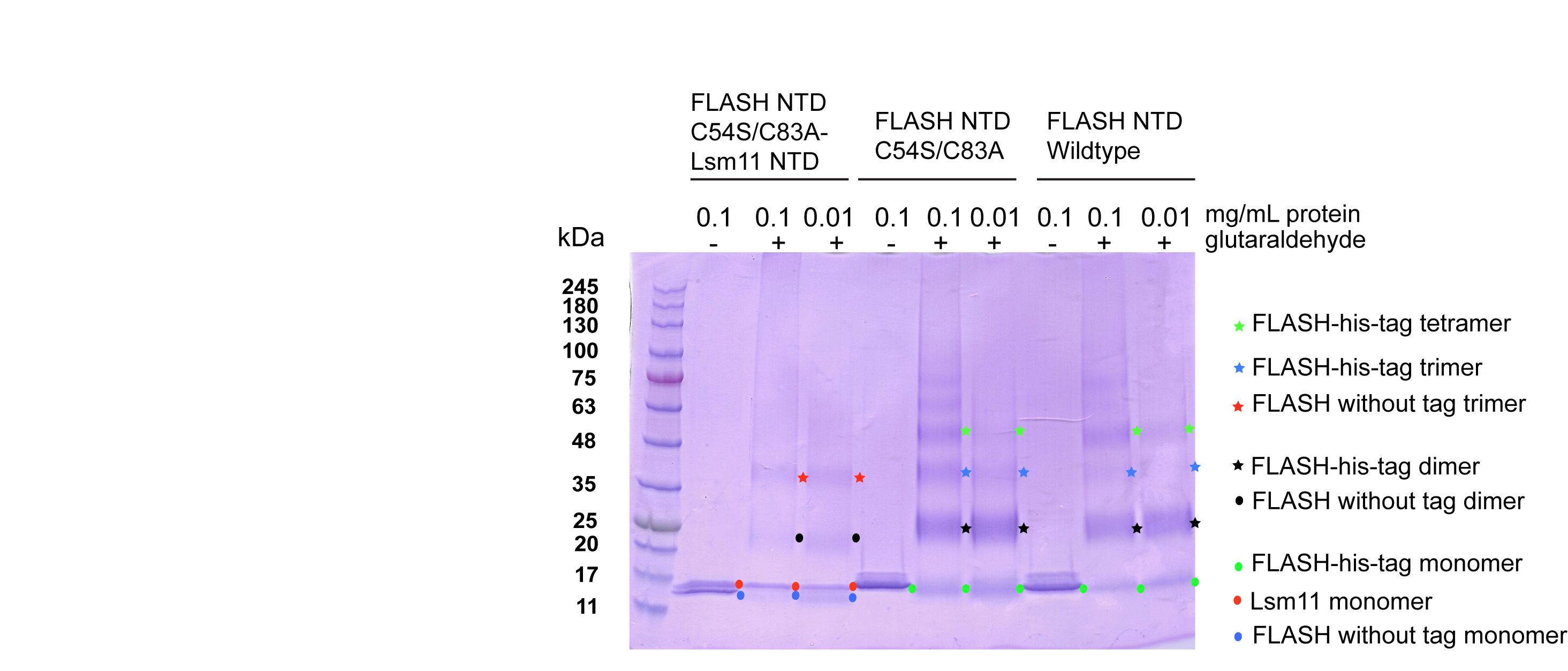

Supplement: S5 Fig — In the FLASH NTD-Lsm11 NTD complex, FLASH NTD is the lower band (as it lacks a His tag compared to FLASH NTD alone), and Lsm11 NTD is the upper band. While the FLASH NTD band disappeared in the presence of glutaraldehyde, the Lsm11 NTD band mostly stayed the same. Therefore, probably very small amount of Lsm11 NTD (if any) got crosslinked in the reaction, consistent with the fact that it has only 1 Lys residue. (TIF) [file pone.0186034.s005.tif]
