# Supplementary material for: The N-terminal domains of FLASH and Lsm11 form a 2:1 heterotrimer for histone pre-mRNA 3’-end processing
Source: PLoS One. 2017 Oct 11;12(10):e0186034. doi: 10.1371/journal.pone.0186034 (PMC5636114; doi:10.1371/journal.pone.0186034)
Supplement: S1 Table — (DOCX) [file pone.0186034.s006.docx]

| Cloning/  Mutagenesis | Plasmid/  Target Construct | Forward primer (5’ to 3’) | Reverse primer (5’ to 3’) | Template for PCR |
| --- | --- | --- | --- | --- |
| Cloning | pET26b-FLASH NTD WT | GTACTACATATGTCAAGAAATTGTTTGGACTTATATG | TACCTCGAGGTGAAGATTACTTATTTCTTCATC | - |
| Cloning | pCDF Duet-FLASH NTD WT | GTACTACATATGTCAAGAAATTGTTTGGACTTATATG | TACCTCGAGTTAGTGAAGATTACTTATTTCTTCATC | - |
| Cloning | pCDF Duet-FLASH NTD C54S/C83A | TTTGTCGACATATGTCAAGAAATAGTTTGGACTTATATG | TGTCGACTCGAGTCAGTGAAGATTACTTATTTCTTCATC | FLASH NTD C54S C83A |
| Mutagenesis | FLASH NTD C54S | ATACATATGTCAAGAAATAGTTTGGACTTATATGAAGAG | CTCTTCATATAAGTCCAAACTATTTCTTGACATATGTAT | FLASH NTD WT |
| Mutagenesis | FLASH NTD C54S/C83A | CAAGTAGAATATGGAAAAGCTCAACTACAAATGAAAGAG | CTCTTTCATTTGTAGTTGAGCTTTTCCATATTCTACTTG | FLASH NTD C54S |
| Mutagenesis | FLASH NTD C54S/C83A/N101A/L104A | AAGTTTAAAGAAATACAGGCACAGGCTTTCAGCGCAATAAACGAAAACCAGTCTCTTAAG | CTTAAGAGACTGGTTTTCGTTTATTGCGCTGAAAGCCTGTGCCTGTATTTCTTTAAACTT | FLASH NTD C54S C83A |
| Mutagenesis | FLASH NTD C54S/C83A/N101A/L104A/ N108A | CAGGCTTTCAGCGCAATAAACGAAGCCCAGTCTCTTAAGAAGAATATTTCAGC | GCTGAAATATTCTTCTTAAGAGACTGGGCTTCGTTTATTGCGCTGAAAGCCTG | FLASH NTD C54S/C83A/N101A/L104A |
| Mutagenesis | FLASH NTD C54S/C83A/R128A/K129A/D130A | CTGCCAGAGTGGAAATAAACGCCGCGGCTGAAGAAATAAGTAATCTTCAC | GTGAAGATTACTTATTTCTTCAGCCGCGGCGTTTATTTCCACTCTGGCAG | FLASH NTD C54S/C83A |
| Mutagenesis | FLASH NTD C54S/C83A/K88A | GGAAAAGCTCAACTACAAATGGCAGAGCTGATGAAAAAGTTTAAAG | CTTTAAACTTTTTCATCAGCTCTGCCATTTGTAGTTGAGCTTTTCC | FLASH NTD C54S/C83A |
| Mutagenesis | FLASH NTD C54S C83A/K88A/K92A/K95A | CTACAAATGGCAGAGCTGATGGCAAAGTTTGCAGAAATACAGGCACAGAATTTC | GAAATTCTGTGCCTGTATTTCTGCAAACTTTGCCATCAGCTCTGCCATTTGTAG | FLASH NTD C54S/C83A/K88A |
| Mutagenesis | FLASH NTD C54S/C83A/Y73A/L76A | GGAACTGCAAAGGAGGCAACAGCTAATGATGCGCAAGTAGAATATGGAAAAGCTC | GAGCTTTTCCATATTCTACTTGCGCATCATTAGCTGTTGCCTCCTTTGCAGTTCC | FLASH NTD C54S/C83A |
| Mutagenesis | FLASH NTD C54S/C83A/Y73A/L76A/Y80A | GCTAATGATGCGCAAGTAGAAGCTGGAAAAGCTCAACTACAAATGAAAG | CTTTCATTTGTAGTTGAGCTTTTCCAGCTTCTACTTGCGCATCATTAGC | FLASH NTD C54S/C83A/Y73A/L76A |
| Mutagenesis | FLASH NTD C54S/C83A/L118A/I119A | CTCTTAAGAAGAATATTTCAGCAGCTGCCAAAACTGCCAGAGTGGAAATAAACC | GGTTTATTTCCACTCTGGCAGTTTTGGCAGCTGCTGAAATATTCTTCTTAAGAG | FLASH NTD C54S/C83A |
